# Genome-Wide Association Study That Identifies Molecular Markers with Freezing Resistance in Duroc Boar Sperm

**DOI:** 10.3390/ani15101474

**Published:** 2025-05-20

**Authors:** Jiajun Zhang, Meicheng Li, Guangxiang Chen, Chenyu Tao, Bushe Li, Hejun Zhang, Hongyang Wang, Wenjun Wang

**Affiliations:** 1 College of Animal Science and Technology, Hebei Agricultural University, Baoding 071000, China; 15630927017@163.com (J.Z.); lmc30574@163.com (M.L.); taochenyuty@163.com (C.T.); 2 Experimental Training Center, Hebei Agricultural University, Baoding 071000, China; chenguang-xiang183@163.com; 3 Shanghai Engineering Research Center of Breeding Pig, Shanghai 201106, China; fordcc@163.com (B.L.); zhj19808@163.com (H.Z.); 4 Key Laboratory of Livestock and Poultry Resources (Pig) Evaluation and Utilization, Ministry of Agriculture and Rural Affairs, Institute of Animal Husbandry &Veterinary Science, Shanghai Academy of Agricultural Sciences, Shanghai 201106, China

**Keywords:** Duroc, GWAS, boar sperm freezability, biomarker, candidate gene

## Abstract

Semen cryopreservation is a crucial technology in the artificial insemination of livestock and poultry. It not only contributes to the conservation of germplasm resources but also facilitates the cross-regional exchange of high-quality breeding stock. In this study, 165 Duroc boars were selected for genome-wide genotyping, and the sperm freezing/thawing motility ratio (sperm recovery rate) was used as phenotypic data for genome-wide association analysis (GWAS). Considerable individual variations in sperm recovery rates (SRRs) were detected, and the sperm structure after cyropreservation was significantly better in highly freeze-tolerant individuals compared to non-freeze-tolerant ones. The heritability of the SRR was calculated and found to be 0.199 ± 0.158, representing low heritability. Through GWAS, eight single-nucleotide polymorphism (SNP) loci and four candidate genes (*SLC10A6*, *MYRF*, *GGA1*, and *UTRN*) were identified as being significantly associated with sperm freezing tolerance. Moreover, the dominant genotypes of four SNPs were finally determined to be valuable for identifying individuals with high sperm freezing tolerance. This study reveals the heritability of the sperm recovery rate and identifies molecular markers associated with sperm freezing tolerance in Duroc boars, which is of great significance for accelerating boar genetic improvement and enhancing the economic efficiency of pig breeding industry.

## Simple Summary

Frozen semen preservation technology is a key technology in the artificial insemination of livestock and poultry and is of great significance in the development of modern animal husbandry. However, it is well known that the freezing ability of boar sperm varies from individual to individual. Therefore, the identification of molecular markers associated with freezing tolerance represents a promising approach to efficiently selecting boars with superior sperm freezing characteristics. In this study, the sperm recovery rate of 165 Duroc pigs was measured as a trait of freezing tolerance for genome-wide association analysis. Through this research, we identified eight SNP loci and four candidate genes that were significantly associated with sperm freezing tolerance. Furthermore, we determined the dominant genotypes of four SNPs, which can potentially be used to identify Duroc boars with high sperm freezing tolerance. Our study provides references and insights into the molecular markers behind the differences in boar sperm freezability and contributes to the advancement of semen cryopreservation technology.

## 1. Introduction

With the increasing spread and normalization of epidemics such as African swine fever, along with the growing demand for the globalized deployment of breeder resources, semen cryopreservation technology has emerged as a key means for safeguarding the security of germplasm resources and overcoming spatial and temporal constraints [1]. However, compared to species like cattle and sheep, porcine sperm exhibit an exceptionally high sensitivity to low-temperature stress. This heightened sensitivity can be attributed to the relatively low cholesterol content in the plasma membrane and the intricate composition of lipids [2]. Consequently, a significant loss of motility occurs after cryo-resuscitation, resulting in a situation in which less than 1% of global boar semen is applied in the form of frozen semen [3]. This limitation significantly restricts the efficient use of high-quality boar genetic resources.

Sperm freezing tolerance is a pivotal biological trait that significantly influences the success of semen cryopreservation. This trait directly impacts the establishment and utilization of genetic resource banks, as well as the efficacy of artificial insemination. It is shaped by a complex interplay of genetic and environmental factors, including seasonal variations, nutrition, breed differences, and the freezing process [4]. Notably, even within the same breed, there are substantial inter-individual variations in sperm tolerance to freezing [5]. To date, several studies have investigated genes related to sperm freezing tolerance. For instance, *STK35* and *IFT27* gene polymorphisms can regulate freezing tolerance by affecting acrosomal structural stability [6]. Genes such as *RNF8*, *PACRG*, and *CADM1* are mainly involved in spermatogenesis, spermatid structural development, and lipid composition, respectively [7]. Additionally, genes associated with inflammation and apoptosis, protein phosphorylation, and energy metabolism, such as *FOS*, *NFATC3*, and *EAF2*, respectively, could serve as markers of freezing tolerance in boars [8]. Despite these advances, conventional approaches to assessing sperm freezing tolerance remain constrained by post-thaw phenotypic evaluations that impose substantial temporal and economic costs, while pre-freezing predictive methodologies continue to present significant technical challenges. The advent of genome-wide association studies (GWAS) has revolutionized genetic marker discovery across agricultural species, successfully identifying loci associated with seminal parameters, including motility-related genes (*PPP2R2B*, *NEK2*, and *NDRG*) [9], concentration-associated markers (*B9D2*, *PAFAH1B3*, *TMEM145*, and *CIC*) [10], and pleiotropic regulators of spermatogenesis (*HIBADH*, *DLG1*, and *MED1*) [11]. Nevertheless, there remain significant gaps in our understanding of the genomic architecture underlying boar sperm freezing tolerance, as current GWAS applications have primarily focused on fresh semen characteristics rather than freeze–thaw resilience. This paucity of molecular data underscores the urgent need for comprehensive investigations to elucidate sperm freezing tolerance-associated genetic variants and establish robust predictive biomarkers for improving sperm preservation protocols.

As the terminal sire breed in the ternary crossbreeding system of the global hog industry, the genetic characteristics of the Duroc pig play an important role in the flesh quality, growth performance, and economic efficiency of commercial pigs [12]. In this study, we calculated the SRR of 165 Duroc boars and identified SNP loci and candidate genes that are significantly associated with the SRR of Duroc boars using GWAS. This work provides a solid foundation for the selection and breeding of highly freeze-tolerant boars. Efforts such as these are important for improving the economic efficiency of pig farms and promoting the healthy development of the pig industry.

## 2. Materials and Methods

### 2.1. Semen Collection

Semen samples were collected from healthy Duroc boars aged 1–3 years at a single breeding farm. All boars are maintained on the same farm, and the temperature and humidity are regulated at appropriate levels. A standardized diet was supplied to ensure consistency in their physiological conditions. Semen samples were obtained from each boar through a minimum of three ejaculations, following a 7-day protocol between collections. Semen collection records with less than 70% sperm motility and those from sick and diseased boars were excluded.

### 2.2. Semen Cryopreservation and Evaluation

Sperm freezing and thawing methods were based on those reported in previous studies [7,13]. Sperm density and motility were measured using a commercial computer-assisted sperm analysis (CASA) system (HVIEW-SSA V8.0, Fuzhou Hong Vision Software Technology Co. (Fuzhou, China)) and recorded. Meanwhile, the straight-line velocity (VSL), curvilinear velocity (VCL), linearity (LIN), wobble (WOB), straightness (STR), beat cross frequency (BCF), average path velocity (VAP), and progressive motility (PR) of the fresh sperm were recorded for subsequent analysis. Fresh semen with ≥70% motility was deemed eligible for cryopreservation. Firstly, the cryo-dilution solution, which was pre-warmed to 37 °C, was added at a ratio of 1:1, gently mixed, and then left to stand at 17 °C for about 2 h. After centrifugation at 800 g/min for 15 min at 17 °C, the supernatant was discarded, leaving the sperm precipitate. Frozen base solution I was added to resuspend the sperm, which was subsequently placed in a 4 °C cryogenic operation cabinet for cooling equilibrium, enabling the semen to slowly cool to 4 °C in 2 h. After equilibration, frozen base solution II was added, mixed well, and immediately canned in a cryogenic cabinet using a semen canning machine to produce 0.5 mL semen tubes. These tubes were placed on a tube rack for 10 min (about 3 cm from the liquid nitrogen surface) and then immersed in a liquid nitrogen tank to complete the preparation of the frozen semen.

One week later, at least three tubes from each sample were selected for thawing. The tubes were thawed in a 50 °C water bath for 16 s. Then, the ends of the tubes were cut and transferred to 5 mL centrifuge tubes. The pre-warmed thawing solution was added to the tubes and recovered in a 37 °C water bath for 15 min to measure the quality of the thawed sperm. The fresh motility/frozen motility ratio of the sperm was calculated and served as the SRR for subsequent analyses.

A total of 577 sperm collection records from 165 Duroc boars were used, and the ratio of sperm fresh motility/frozen motility was calculated as the SRR for subsequent analyses.

### 2.3. Structural Testing of Sperm

The integrity of the sperm plasma membrane was assayed using the Hoechst 33342/PI double staining kit (CA1120, Solarbio, Beijing, China). For each sample, 20 μL of semen was carefully pipetted and evenly coated on a glass slide, and then the operations were strictly carried out according to the kit instructions. Under the fluorescence microscope, sperm with an intact plasma membrane exhibited blue fluorescence, whereas sperm with a damaged plasma membrane showed red fluorescence.

The mitochondrial membrane potential was assayed using the JC-1 kit (J8030, Solarbio, Beijing, China). For each sample, 500 μL of semen was taken, and 1000 μL of phosphate-buffered saline (PBS) was added. After centrifugation, the supernatant was discarded, and the precipitate was resuspended in 400 μL of PBS. Subsequently, it was mixed with 0.5 mL of JC-1 working solution and incubated at 37 °C for 20 min. At the end of the incubation period, the sample was washed twice with JC-1 staining buffer (4 °C, 1000× *g*, centrifugation for 6 min). Under the fluorescence microscope, sperm with a high mitochondrial membrane potential displayed red fluorescence, while those with a low mitochondrial membrane potential showed green fluorescence.

The acrosome integrity assay was performed using the FITC-PSA kit (Anhui Anke Biotechnology Co., Ltd., Hefei, China). For each sample, 500 μL was taken, and 1000 μL of PBS was added. After centrifugation at room temperature, 1000× *g*, for 6 min), the precipitate was resuspended in 100 μL of PBS, Then, 5 μL of the mixed suspension was added to the cell staining fixative, followed by the addition of 5 μL of FITC-PSA. The mixture was incubated at room temperature for 30 min in the dark for the assay. Under fluorescence microscopy, it was observed that sperm with an intact acrosome presented with bright green, evenly distributed acrosomes, while sperm with an incomplete acrosome showed different degrees of fluorescence deficiency or irregular staining patterns.

For the statistics of sperm plasma membrane integrity, mitochondrial membrane potential, and acrosome integrity indices, at least five clear fields of view, each containing at least 200 sperm, were selected.

### 2.4. Genotype Data Acquisition and Quality Control

A total of 165 Duroc semen samples were sent to Beijing Compass Biotechnology Co., Ltd. (Beijing, China) for DNA extraction. Subsequently, they were genotyped using a custom 50K SNP chip (“Zhongxin No. 1”), which was designed in the Infinium XT-96 format (Compass Biotechnology, Beijing, China) [14,15]. Quality control criteria were as follows: (1) SNPs located at unknown positions and those on sex chromosomes were excluded; (2) individuals with an SNP detection rate lower than 90% were removed; (3) SNPs with a detection rate of less than 90% were excluded; (4) SNPs with a minor allele frequency below 0.05 were filtered out; and (5) SNPs deviating from the Hardy–Weinberg equilibrium test with a *p*-value less than 10^−4^ were discarded.

### 2.5. Estimation of Heritability and Population Affinity Coefficients for SRRs

In this experiment, linear mixed models were used to estimate heritability by fitting the effects of all SNPs as random effects. The models were formulated as follows:

y = WW′σ^2^ u + Iσ^2^ε (1)

where y denotes the vector of phenotypes for resuscitation rates; u denotes the vector of SNP effects, obeying the following distribution: u~N (0, Iσ^2^u); W denotes the standardized genotype matrix; I denote the n × n unit matrix; and ε denotes the vector of residual effects.

The genotype matrix data from the microarray were converted into a gene content matrix. Specifically, the parameters are expressed as the number of minimum frequency alleles in the genotype, namely, 0, 1, and 2. Here, 0 denotes major frequent (uppercase letters) pure heterozygotes, 2 denotes minor frequent (lowercase letters) pure heterozygotes, and 1 denotes heterozygotes.

### 2.6. Genome-Wide Association Analysis of Semen Freezing Tolerance

In this study, GWAS was conducted on microarray data using Tassel 5.0 software. A genome-wide association analysis significance threshold of 3 × 10^−5^ (1/36,430) and a suggestive threshold of 6 × 10^−4^ were determined using the Bonferroni method.

Genetic markers were detected using a mixed linear model, which was formulated as follows:

y = Xα + Zβ + Wμ + e(2)

where y denotes the SRR phenotype vector; X denotes the marker effect matrix, with effect values corresponding to SNPs; α denotes the regression coefficient for marker effects, indicating the fixed effect of each SNP on SRR; Z denotes the population genetic background matrix, which is used to control the top three principal component effects of the population genetic background; β denotes the regression coefficient for principal component effects; W denotes the kinship matrix kinship, referring to the kinship within the population; μ denotes the regression coefficient of the kinship matrix; and e denotes the model residuals.

### 2.7. Statistical Analyses

Tukey’s multiple comparison association analysis was performed using the lme4 program package (https://github.com/lme4/lme4, accessed on 2 July 2024) in RStudio (version 4.3.2) for sperm recovery rate traits and the significant SNP loci screened using GWAS (version 1.1-37). The purpose of this analysis was to identify genotypes with significant differences (*p* < 0.05). Then, SNP density maps were generated using the CMPlot (version 4.5.1) package (https://github.com/YinLiLin/CMplot, accessed on 26 July 2024) in RStudio. Moreover, the Ldheatmap package (version 1.0-6) (https://github.com/SFUStatgen/LDheatmap, accessed on 15 August 2024) in RStudio was used to analyze the linkage disequilibrium of SNP loci and plot the linkage disequilibrium decay map. The SNP effect values and correlations between SNPs and phenotypes were tallied using the ggplot2 (version 3.5.2) (https://github.com/tidyverse/ggplot2, accessed on 20 August 2024) and pheatmap program packages (https://github.com/raivokolde/pheatmap, accessed on 26 September 2024) in RStudio. The population structure of Duroc pigs was analyzed using Structure (version 2.3.4).

## 3. Results

### 3.1. Sperm Motility and Structure Differ Before and After Freezing

**Figure 1:**
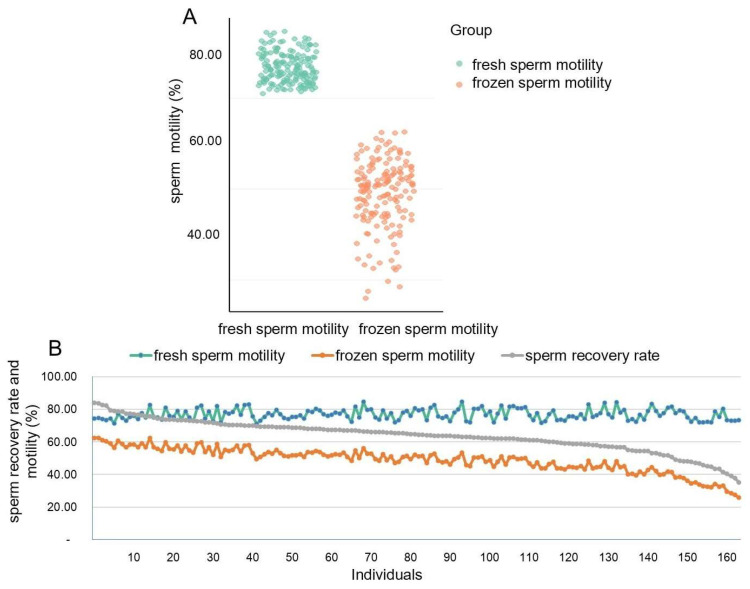
Fresh and frozen sperm motility in Duroc boars. (**A**) Scatter plot of fresh and frozen sperm motility in Duroc boars; (**B**) Fresh sperm motility, frozen sperm motility, and recovery rate of 165 Duroc boars.

**Figure 2:**
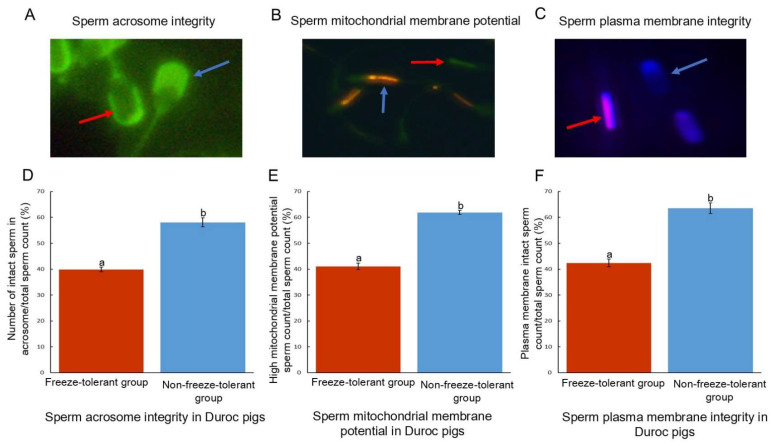
Structural staining and statistical analysis of sperm. (**A**) Sperm acrosome integrity; (**B**) Sperm mitochondrial membrane potential; (**C**) Sperm plasma membrane integrity; (**D**) Statistical analysis of sperm acrosome integrity in freeze-tolerant and non-freeze-tolerant individuals; (**E**) Statistical analysis of mitochondrial membrane potential of sperm in freezing-tolerant and non-freezing-tolerant individuals; (**F**) Statistical analysis of plasma membrane integrity of sperm in freezing-tolerant and non-freezing-tolerant individuals. Note: Different small letter superscripts indicate significant differences (*p* < 0.05).

The average motility of fresh sperm was 76.8 ± 3.34%, while that of frozen sperm was 48.9 ± 6.56%, with the corresponding coefficients of variation being 4.35% and 13.42%, respectively (Figure 1A). To assess sperm freezing tolerance, the SRR was calculated for each individual, revealing significant individual variability in sperm freezing tolerance (Figure 1B). The top 20% and bottom 20% of SRR values were selected as the freezing-tolerant group and non-freezing-tolerant group, respectively. Subsequent analysis showed that sperm in the freezing-tolerant group exhibited superior acrosome integrity, plasma membrane integrity, and mitochondrial membrane potential compared to those in the non-freezing-tolerant group (Figure 2). These findings suggest that sperm from individuals with different freezing tolerances experience varying degrees of structural damage.

### 3.2. Correlation Analysis of Sperm Motility Parameters

**Figure 3:**
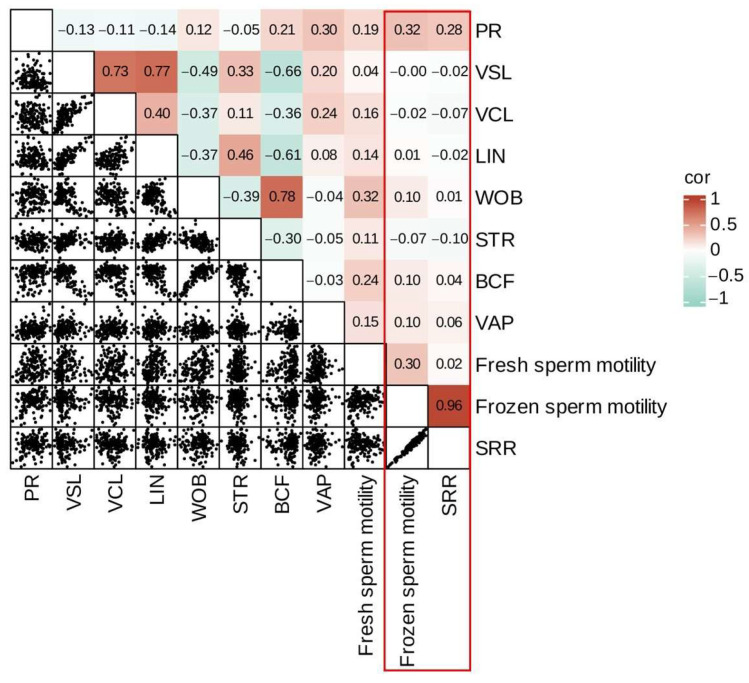
Correlation analysis of fresh sperm motility and frozen sperm motility parameters.

To identify the indicative traits of boar sperm freezing tolerance prior to semen cryopreservation, a correlation analysis was performed among fresh sperm motility, frozen sperm motility, and the SRR (Figure 3). The results showed that frozen sperm motility was weakly positively correlated with PR and fresh sperm motility (*p* < 0.01), while it had no correlation with VSL, VCL, LIN, WOB, STR, BCF, or VAP (*p* > 0.05). Moreover, a statistically significant but weak positive correlation was observed between the SRR and PR (r = 0.28, *p* < 0.01), suggesting that relying solely on PR is inadequate for accurately predicting the SRR in the context of semen cryopreservation. These findings imply that it is not feasible to reliably predict the post-thaw motility of sperm based on specific parameters of fresh sperm prior to semen cryopreservation.

### 3.3. Population Genetic Structure Analysis and Heritability Estimation of the SRR

**Figure 4:**
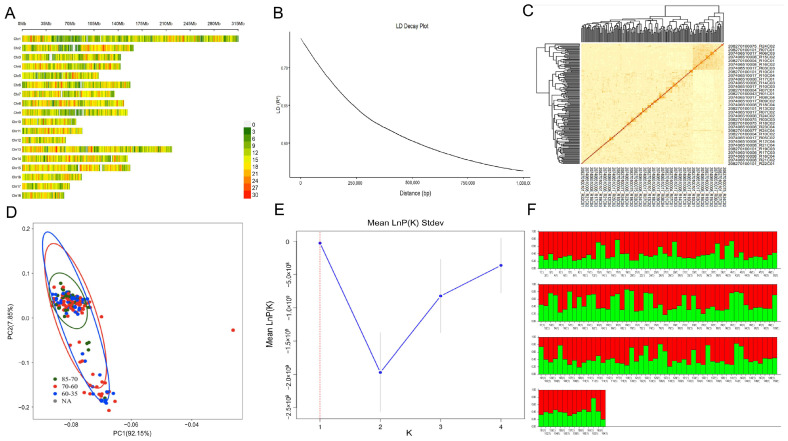
Population genetic structure analysis of Duroc populations. (**A**) Distribution of SNPs on chromosomes after quality control; (**B**) LD attenuation diagram; (**C**) Heatmap of kinship; (**D**) PCA analysis of the SRR; (**E**) Predicting the optimal K value; (**F**) Group structure of Duroc pigs.

Following quality control, a total of 36,430 SNP loci from 164 Duroc boars were identified, exhibiting a relatively uniform distribution across the chromosomes (Figure 4A). Additionally, these SNPs provided adequate genome-wide coverage (Figure 4B). The average genomic kinship coefficient for Duroc pigs was 0.082 ± 0.147, indicating that the Duroc pig population as a whole is relatively distant and possesses a relatively homogeneous genetic structure within the population (Figure 4C,D). The genetic structure of the 164 Duroc populations after quality control was analyzed using Structure software (version 2.3.4) to determine the optimal K value (K = 2) (Figure 4E). All individuals were divided into two subpopulations (red and green), indicating that the present experimental population hybridized from two ancestral subpopulations (Figure 4F). These results collectively suggest that the population does not possess a more complex genetic structure and remains stable.

To estimate the heritability of the SRR, a linear mixed model was employed. The heritability of SRR was calculated to be 0.199 ± 0.158, indicating low heritability. This indicates that the SRR trait has a moderate degree of heritability within the porcine population.

### 3.4. Genome-Wide Association Analysis and Candidate Gene Identification

**Figure 5:**
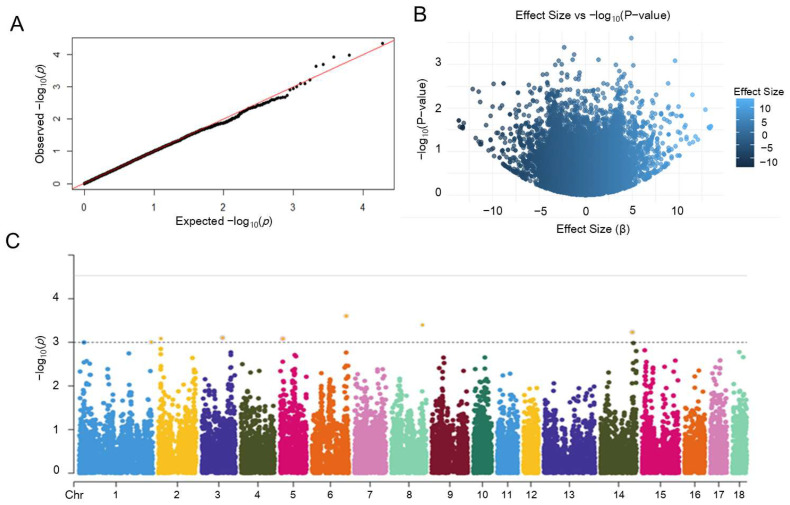
Key visualizations of GWAS for the SRR. (**A**) Quantile–Quantile plot of GWAS results for SRR; (**B**) Scatter plot of SNP effect sizes vs. −log_10_(*p*-values); (**C**) Manhattan plot identifying significant loci associated with SRR.

GWAS was performed to identify loci associated with SRR in Duroc boars. The Quantile–Quantile plot demonstrated that observed chi-square statistics of SNPs aligned closely with expected values, indicating effective control of population stratification (Figure 5A). Analysis of SNP effect sizes versus −log_10_(*p*-values) revealed eight SNPs showing substantial effects (|β| > 2) and strong significance (*p* < 0.005) located in the upper-right quadrant (Figure 5B). These significant SNPs passed the Bonferroni-corrected threshold (3 × 10^−5^) and were distributed across seven chromosomes (Chr 1, 2, 3, 5, 6, 8, and 14), as visualized in the Manhattan plot (Figure 5C).

Through the integration of published literature and functional annotation, four candidate genes (*SLC10A6*, *MYRF*, *GGA1*, and *UTRN*) corresponding to and flanking (<1 Mb) each significantly associated SNP were identified (Table 1). These genes are functionally implicated in biological processes, including gonadal development and spermatogenesis, which could be used as candidate genes that affect sperm freezing tolerance in Duroc boars.

### 3.5. Determination-Dominant Genotypes Associated with SRR Traits

A multiple comparison analysis of eight SNP loci significantly associated with the SRR trait in Duroc pigs revealed that five SNPs (CNC10050191, CNC10062936, CNC10142631, CNC10031720, and CNC10031721) exhibited significant differences in SRR trait expression (*p* < 0.05) (Table 2). Further investigation identified CC, AA, AG/GG, and AG/GG as the dominant genotypes for SNPs CNC10050191, CNC10062936, CNC10031720, and CNC10031721, respectively (*p* < 0.05) (Table 3). These findings establish a methodological framework for the subsequent screening of Duroc populations to identify individuals with high sperm freezing tolerance.

## 4. Discussion

Sperm freezing tolerance refers to the ability of sperm to maintain viability, motility, and fertilization function after freezing–thawing. This ability reflects the inherent biological characteristics of sperm related to resisting low-temperature-induced damage. By using the SRR as the core evaluation index, it becomes possible to quantify the proportion of sperm that survive after freezing. Additionally, this index can visually represent the overall maintenance of sperm plasma membrane integrity, mitochondrial function, and motility. Cryopreservation induces structural damage, such as plasma membrane rupture, the loss of mitochondrial membrane potential, and acrosome breakdown through ice crystal formation, as well as osmotic pressure imbalance and oxidative stress [16,17]. The disruption of plasma membrane integrity gives rise to osmoregulatory failure and energy metabolism disruption, thereby diminishing the efficacy of cryoprotectants [18]. The accumulation of reactive oxygen species (ROS) and impaired ATP synthesis reduce sperm motility [19]. Moreover, damage to the sperm acrosome membrane structure affects the loss of sperm function during the release of key enzymes that penetrate the zona pellucida of the egg [20]. It has been demonstrated that sperm from individuals with high freezing tolerance have significantly better plasma membrane integrity, mitochondrial function, and acrosome integrity compared to those from individuals with low freezing tolerance [7]. This finding validates that sperm structural integrity serves as the foundation for freezing tolerance. Therefore, the SRR assay not only enables the rapid screening of individuals with high freezing tolerance but also provides a theoretical basis for optimizing sperm anti-freezing damage strategies by evaluating the indicators of plasma membrane integrity, mitochondrial function, and acrosome integrity.

Heritability estimation plays a pivotal role in the scientific formulation of breeding programs, precise prediction of selection responses, and vigilant monitoring of population genetic progress [21]. A high heritability value signifies that the genetic contribution to a trait is substantial, and the trait can be significantly altered via selective breeding. Conversely, low heritability indicates that the trait is predominantly influenced by environmental factors, thereby limiting the potential for improvement through genetic selection [22]. The heritability of SRR is affected by the combined effect of multiple genes in the genome. There are relatively few studies on the heritability of SRR, but it has been shown that the heritability of sperm motility varies among species and breeds [23,24]. Semen samples from 2617 bulls were utilized, and the estimated heritability of sperm motility was found to be 0.09 [25]. Another study analyzed semen from 1450 Chinese Holstein bulls with complete pedigree information. After calculating the heritability of several traits, the heritability of sperm motility was determined to be 0.12 [26]. For Holstein bulls, the heritability of sperm motility was estimated to be 0.16 [27]. In Ethiopian sheep, the heritability of the sperm motility trait in 9-month-old and 12-month-old rams were found to be 0.32 and 0.27, respectively [28]. In the case of Holstein bulls, the heritability was found to be 0.43 [29]. The results of this study demonstrated that the SRR of Duroc boars is a trait with low to medium heritability, specifically 0.199 ± 0.158. Given that the current heritability of SRR is not particularly high, relying solely on selecting for a high recovery rate may not be entirely reliable for breeding purposes. However, with the aid of molecular breeding techniques and genetic marker-assisted selection, it is feasible to conduct more refined selection based on specific genes. By performing genome-wide association analysis on recovery rate traits to screen for molecular markers that may influence the SRR and using these markers to assist in the selection of individuals with superior recovery rates, the effectiveness of sperm cryopreservation can be significantly enhanced.

In this study, we identified four genes that are potential key candidates for influencing sperm freezing tolerance in Duroc boars. The sodium-dependent organic anion transporter SOAT (SLC10A6), a plasma membrane transporter of sulfated steroids, is highly expressed in germ cells of the testis [30]. SOAT transfers biologically inactive sulfated steroids to specific target cells, where they are reactivated by steroid sulfatase to become a biologically active unconjugated steroid that is known to regulate spermatogenesis [31,32]. In humans, *SOAT* is predominantly expressed in the testis and is also detectable in the skin, placenta, pancreas, and mammary glands [26]. In mice, *SOAT* expression is highest in the lungs, skin, and testis, with the order of expression depending on the mouse strain analyzed (CF-1, C57BL/6, FVB, or 129Sv) [33]. In all of these mice, *SOAT* proteins were localized to spermatocytes and spermatids, thus speculating on the potential regulatory role of this vector for sperm freezing tolerance. The myelin regulator encoded by the *MYRF* gene is a membrane-bound transcription factor that can be cleaved by proteolytic hydrolysis into its C-terminal and N-terminal structural domains [34]. The C-terminal fragment stays in the endoplasmic reticulum, while the N-terminal region, which contains the DNA-binding domain, enters the nucleus and thus promotes target genes. A previous study has revealed that *MYRF* is expressed in a variety of tissues, including the heart, lung, diaphragm, and eye, as well as the somatic epithelium of the fetal gonad [35]. Additionally, *MYRF* was highly expressed in the corpora cavernosa epithelium (CE)/corpus cavernosa epithelium-derived cells (CEDC) of human and mouse fetal gonads [36]. Gene ontology (GO) enrichment analysis of potential *MYRF* target genes showed significant enrichment of GO terms related to migration and proliferation among them. Therefore, we hypothesized that the loss of *MYRF* function may lead to the defective development of the germinal epithelium, which in turn interferes with sperm environmental homeostasis, allowing for structural changes in porcine sperm that reduce resistance to damage during freezing. *GGA1* is a Golgi-associated protein-coding gene. In drosophila, it is associated with spermatogenesis, but its function in mammalian spermatogenesis remains unclear [37]. A study has shown that *GGA1* knockout mice exhibit severe disruption in spermatogenesis and become infertile when exposed to bisphenol A, suggesting that *GGA1* plays an important role in spermatogenesis under stress [38]. The Golgi apparatus is responsible for the formation of the acrosome during spermatogenesis [39]. During the early stages of spermatogenesis, small vesicles produced by the Golgi apparatus accumulate in the concave region of the sperm nucleus, and as the sperm develop, the small vesicles fuse to form large acrosomal particles [40]. Thus, *GGA1* may be involved in the spermatogenesis process in boars by participating in the transportation of Golgi vesicles. Myostatin (a product of UTRN) is the autosomal homolog of antimyostatin. Myostatin and antimyostatin may affect sperm motility and play a role in the sperm flagellum through different mechanisms [41]. It has been demonstrated that anti-myosin/myosin is required to regulate the balance between proliferation and apoptosis during spermatogenesis. By detecting the reduction of Ki67-positive and microtubule-associated protein MARK2-positive spermatogenic cells, it has been shown that the lack of myostatin and endometrial atrophin leads to the proliferation arrest of spermatogenic cells, which provides a basis for resolving the regulatory network of *UTRN* in freezing tolerance. All these four genes are worthy of our validation of their roles in the freezing tolerance of boar sperm through gene knockout and/or other methods. In addition, based on the dominant genotypes of the candidate SNP loci, we screened 307 alternative Duroc boars and identified 66 candidate boars with high freezing tolerance. The semen of these boars will be frozen and tested after they reach sexual maturity to confirm the freezing tolerance of the selected individuals. Moreover, sperm cryoresistance is a sex-limited trait that can only be phenotypically assessed in boars. In most commercial pig production systems, the inherently limited number of breeding boars leads to a relatively small effective population size for such studies, which fundamentally constrains the statistical power of genetic analyses. Although we included multiple ejaculates per individual to increase phenotypic accuracy and reduce measurement error, the restricted number of animals remains a major challenge. Future research should consider expanding the sample size by incorporating boars from multiple herds, breeding lines, or collaborative programs.

## 5. Conclusions

In the present study, GWAS was conducted to identify SNPs associated with SRR in Duroc boars. A total of eight significant SNPs were identified through GWAS. Further analysis revealed that four genes, namely, *SLC10A6*, *MYRF*, *GGA1*, and *UTRN*, were associated with these SNPs and were implicated in gonadal development and spermatogenesis. Additionally, the dominant genotypes of the four most significant SNPs were determined. These findings provide a scientific basis for improving freezing tolerance in pig populations in the future. The process of the genetic improvement of pigs can be accelerated through molecular marker-assisted selection based on the identified genetic markers.

## Figures and Tables

**Table 1:** Important candidate genes that affect the freezing tolerance of sperm.

Significant SNP	Candidate Genes	Gene Position	Biological Process
CNCB10006390	*SLC10A6*	8:131997359-132023739	Spermatogenesis
CNC10020205	*MYRF*	2:9761230-9795873	Gonadal development
CNC10050191	*GGA1*	5:10186132-10213830	Spermatogenesis
CNC10015560	*UTRN*	1:20514264-21047927	Spermatogenesis

**Table 2:** Multiple comparison analysis of genotypes with SRR in Duroc boars.

SNP Name	Genotypes	Number	*p* Value	Mean SRR ± SD (%)
CNC10015560	TT	59	0.298339167	65.0783 ± 9.2839
	CT	74		62.5943 ± 9.1071
	CC	31		63.5574 ± 11.0034
CNC10020205	AA	14	0.068627451	64.1842 ± 9.9289
	AG	75		61.8949 ± 9.1276
	GG	75		65.3490 ± 9.7195
CNC10050191	TT	4	0.002646241	48.4825 ± 10.2948 ^b^
	TC	31		62.6477 ± 9.9919 ^a^
	CC	129		64.3865 ± 9.0806 ^a^
CNCB10006390	TT	50	0.267082987	65.0460 ± 9.7249
	TC	77		62.3490 ± 9.7412
	CC	37		64.5594 ± 8.7953
CNC10062936	GG	32	0.007107671	59.6181 ± 9.8161 ^b^
	GA	86		63.8147 ± 9.4879 ^a^
	AA	46		66.2180 ± 8.7233 ^a^
CNC10142631	AA	64	0.046342115	65.2662 ± 9.9858 ^a^
	GA	71		61.3832 ± 9.6985 ^b^
	GG	29		65.7458 ± 6.9775 ^a^
CNC10031720	AA	38	0.016191411	60.1160 ± 11.5879 ^b^
	AG	82		65.2585 ± 9.2777 ^a^
	GG	44		63.7788 ± 7.2515 ^a^
CNC10031721	GG	38	0.016191411	60.1160 ± 11.5879 ^b^
	GA	82		65.2585 ± 9.2777 ^a^
	AA	44		63.7788 ± 7.2515 ^a^

Note: For each locus, values with no letter superscripts indicate no significant difference (*p* > 0.05), while those with different small letter superscripts indicate significant differences (*p* < 0.05).

**Table 3:** Dominant genotypes for boar SRR.

SNP Name	CNC10050191	CNC10062936	CNC10031720	CNC10031721
Dominant genotype	CC	AA	AG/GG	GA/AA

## Data Availability

Data will be made available upon request.

## Abbreviations

The following abbreviations are used in this manuscript:

GWAS	Genome-wide association study
SNP	Single-nucleotide polymorphism
SRR	Sperm recovery rate
VSL	Straight line velocity
VCL	Curvilinear velocity
LIN	Linearity
VAP	Average path velocity
WOB	Wobble
STR	Straightness
BCF	Beat cross frequency
PR	Progressive motility
